# The Effects of Aerobic Exercise Training on Testosterone Concentration in Individuals Who are Obese or Have Type 2 Diabetes: A Systematic Review and Meta-Analysis

**DOI:** 10.1186/s40798-024-00781-x

**Published:** 2024-10-29

**Authors:** Rhiannon Healy, Rhiannon Patten, Carlie Bauer, Mary N. Woessner, Matthew Bourke, Mathis Grossmann, Itamar Levinger

**Affiliations:** 1 Institute for Health and Sport (IHES), Victoria University, PO Box 14428, Melbourne, Australia; 2 School of Human Movement and Nutrition Sciences, The University of Queensland, Brisbane, QLD Australia; 3 University of Melbourne Austin Health, Heidelberg, VIC Australia; 4 Australian Institute for Musculoskeletal Science, University of Melbourne and Western Health, Melbourne, VIC Australia

**Keywords:** Aerobic exercise, Testosterone, Obesity, Type 2 diabetes, Sex hormone binding globulin, Insulin sensitivity

## Abstract

**Background:**

Obesity and type 2 diabetes (T2D) are associated with alterations in testosterone concentrations. While evidence indicates that aerobic training can influence testosterone in healthy populations or females with hyperandrogenism, its impact in individuals with obesity or T2D remains unclear. Thus, the aim of this study was to investigate whether aerobic training can influence circulating testosterone concentrations in individuals with obesity or T2D.

**Methods:**

EBSCOhost (CINAHL, MEDLINE, SPORTDiscus), PubMed and Embase were searched for articles published until August 2023. Eligible articles included individuals with obesity or T2D that underwent an aerobic exercise intervention with testosterone concentrations measured at baseline and post intervention. Two reviewers independently screened the seven articles included in this meta-analysis and conducted data extraction and risk of bias assessments.

**Results:**

A total of 103 participants (62 men / 41 women) from three randomised controlled trials and four non-randomised controlled trials were included. Effect sizes were computed with random effects models. Aerobic exercise moderately increased testosterone concentrations in men (g = 0.565, 95% CI = 0.307 to 0.822, *p* < 0.001), but had no significant effect in women (g = -0.523, 95% CI = -1.541, 0.496, *p* = 0.314). Aerobic exercise had no significant effect on sex hormone-binding globulin or markers of insulin sensitivity (*p* > 0.05).

**Conclusions:**

Aerobic training may be used to increase testosterone concentrations in men with obesity or T2D, but potentially has no influence in women. Given the low number of studies, further studies investigating the effect of exercise on circulating sex hormones in men and women with obesity or T2D are needed.

## Key Points

• Aerobic exercise training can significantly increase testosterone concentrations in men who are obese or have type 2 diabetes.

• Aerobic exercise training does not appear to significantly influence testosterone concentrations in women who are obese or have type 2 diabetes.

• Aerobic exercise training could be utilised to improve testosterone concentrations in men who are obese or have type 2 diabetes.

## Background

The prevalence of obesity and type 2 diabetes (T2D) continues to grow worldwide. In developed counties, it is estimated that 60% of the adult population are overweight or obese and up to 7% live with T2D [1–3]. Among other factors, changes in circulating sex steroid concentrations, in particular testosterone, have been associated with the progression of obesity and T2D [4–6].

Testosterone plays an important role in both reproductive and non-reproductive systems [7, 8]. It is produced via the gonads following stimulation by gonadotropic hormones and circulates in the blood as either free or bound to albumin or carrier glycoproteins, including sex hormone-binding globulin (SHBG) [9, 10]. Testosterone has been reported to influence metabolism and insulin sensitivity in both men and women, with low and high levels being associated with negative health consequences in men and women, respectively [9, 11]. Low concentrations of testosterone in men have been associated with increased incidence of T2D, obesity, hypertension, and hypercholesterolemia [4, 5, 12–15], while high concentrations of testosterone in women have been associated with higher body fat mass, risk of T2D and insulin resistance (IR) [16–18]. This inverse association with disease risk between sexes is likely due to the differing primary sex hormones in men and women, with undesirable changes to basal ranges resulting in negative health consequences [7].

Current treatment for abnormal testosterone concentrations includes lifestyle interventions or pharmacological therapies [19–21]. Lifestyle interventions aim to achieve a healthy body weight through exercise, diet or a combination of the two [19]. Pharmacological therapies, such as testosterone replacement therapy or combined oral contraceptives, use bioidentical or synthetic hormones to treat organic hypogonadism or hyperandrogenism in men and women, respectively [22, 23]. Hormone replacement therapies pose a greater risk for side effects than lifestyle interventions, such as risk of erythrocytosis, infertility, and weight gain, and have conflicting results on their efficacy in reducing the disease risk associated with obesity and T2D [22, 24–27]. Conversely, lifestyle interventions, particularly those utilising exercise, are an easily accessible and non-pharmacological approach to lower disease risk with minimal adverse side effects [28, 29]. There is also some evidence to suggest that aerobic exercise training can increase testosterone concentrations in healthy active populations [30–32] while also decreasing testosterone concentrations in women with hyperandrogenism as a result of polycystic ovary syndrome [33]. Aerobic exercise has been found superior to caloric restriction for improving testosterone concentrations in men who are overweight or obese [34], and been suggested to induce greater improvements in cardiovascular health compared to resistance exercise [35], making it an ideal intervention for improving both hormonal and cardiovascular health. However, the evidence on aerobic exercise and testosterone in individuals who are obese or have T2D is limited.

Thus, the aim of this study was to identify and quantitatively synthesise existing literature on the effect of aerobic exercise training on circulating testosterone concentrations in men and women who are obese or have T2D. It is hypothesised that aerobic exercise training will increase concentrations of circulating testosterone in men and reduce concentrations of circulating testosterone in women. The study also aimed to investigate the effects of aerobic exercise training on SHBG and markers of insulin sensitivity in individuals who are obese or have T2D.

## Methods

### Protocol and Registration

This systematic review and meta-analysis were conducted and reported in accordance with the Preferred Reporting Items for Systematic Reviews and Meta-Analyses (PRISMA) and was registered on the International prospective register for systematic reviews (PROSPERO; CRD42023412043).

### Search Strategy, Study Selection and Data Extraction

A systematic search of the literature was conducted from inception to August 2023, using EBSCOhost (CINAHL, MEDLINE, SPORTDiscus), PubMed and Embase. The following search strategy utilised: keyword and categorical searches (i) ‘insulin resistance’ OR ‘insulin resistan*’ OR obese OR obesity OR ‘type 2 diabet*’ OR ‘diabet*’ OR T2D* OR ‘type 2 diabetes’ OR overweight OR ‘impaired glucose tolerance’ (ii) ‘Aerobic exercise’ OR ‘chronic exercise’ OR ‘acute exercise’ OR ‘high intensity interval’ OR ‘aerobic training’ OR ‘high-intensity-interval’ OR HIIT OR high-intensity intermittent OR high intensity intermittent (iii) Testosterone OR androgen* OR ‘reproductive hormon*’ OR ‘sex hormone’ OR ‘steroid hormon’ OR dihydrotestosterone OR ‘gonadal steroid hormones’ OR ‘gonadal hormones’ OR ‘gonadal hormone*’ (iv) NOT ‘deprivation therapy’ or ‘replacement therapy’ or cancer. Categories i – iii were combined using ‘AND’. After removal of duplicates, all articles were reviewed and full-text screened independently by two authors. Any discrepancies were resolved by consensus. After full-text screening, data extraction was completed by RH, RP and CB using a pre-determined extraction form.

Where required, authors were contacted via email using an institutional email to obtain additional or raw data when necessary. Following a second email, if no response was received within 14 days, the article was excluded from the meta-analysis. Where multiple publications resulted from the same trial, results were combined, and only one result (largest participant number) for each outcome was used in the analysis.

### Eligibility Criteria

The Population, Intervention, Comparison, Outcomes and Studies (PICOS) framework was used for this meta-analysis (Table 1). Studies which met the predefined criteria were included. In brief, included studies had participants over the age of 18 years with either obesity or T2D. Obesity was determined by a body mass index (BMI) of > 30 kg/m^2^ or > 27.5 kg/m^2^ for participants of an Asian descent [36]. Studies with a definition of obesity as a high body fat percentage with a BMI < 27.5 kg/m^2^ were not included. An exception was made in one instance where the mean BMI was 0.1 kg/m^2^ below the relative ethnic BMI cut-off [37]. Participants with T2D were included regardless of BMI.

The intervention included any aerobic exercise intervention, regardless of intensity, that was greater than two weeks in duration to ensure the effects measured were reflective of exercise training and not transient changes in testosterone as a result of a single exercise bout. Studies with concurrent exercise interventions or dietary/supplement interventions were excluded.

The primary outcome specified for this meta-analysis was serum total testosterone changes following an aerobic exercise intervention, and therefore only studies which measured total serum testosterone pre and post aerobic exercise intervention were included.

Secondary outcomes included free testosterone, oestradiol, SHBG, dehydroepiandrosterone (DHEA), follicle stimulating hormone (FSH), luteinising hormone (LH), fasting blood glucose (FBG), fasting insulin, quantitative insulin-sensitivity check index (QUICKI), homeostatic model assessment for insulin resistance (HOMA-IR) and hemoglobin A1c (HbA1c). Studies were only eligible for analysis of secondary outcomes if they also reported on the primary outcome.

**Table 1:** Eligibility criteria for study inclusion

Participant	Intervention	Comparator	Outcome	Study Design	Limits
*Inclusion criteria*: - Adults over 18 years of age; - Diagnosed T2D; OR - Obesity. As established by a mean BMI of > 30 kg/m^2^ or > 27.5 kg/m^2^*.	*Inclusion criteria*: - Any aerobic exercise intervention. *Exclusion criteria*: - Muscle strengthening / concurrent exercise; - Concurrent diet / supplement intervention; - Hormone replacement therapy.	Hormone concentrations prior to completing the exercise intervention.	Primary outcome: Total serum testosterone concentration. *Secondary outcomes*: - Sex hormone concentrations (oestradiol, SHBG, DHEA, FSH, LH); - Measures of insulin sensitivity and glucose regulation.	All study designs.	English language; Human participants; Peer reviewed; Published.

*Asia-Pacific classification; T2D: type 2 diabetes; BMI: body mass index; SHBG: sex hormone-binding globulin; DHEA: dehydroepiandrosterone; FSH: follicle stimulating hormone; LH: luteinising hormone

### Assessment of Risk of Bias in Included Studies

The Risk of Bias assessment tool 2 (RoB2) was used to evaluate the included randomised controlled trials (RCTs) [38]. The risk of bias in non-randomised studies – of interventions (ROBINS-I) was utilised to assess risk of bias in non-randomised controlled trials (CTs) [39]. Three reviewers (RH, CB, MW) independently assessed the methodological quality and disagreements were resolved by consensus.

### Statistical Analysis

#### Calculation of Effect Sizes

The standardised mean difference (SMD; Hedges’ g) in testosterone, SHBG and insulin sensitivity markers from pre-to-post exercise intervention were used as the effect sizes in the meta-analysis. The SMD was calculated as the difference in the pre and post exercise means of each outcome, divided by the standard deviation of change, corrected for upward bias of small sample sizes. The standard deviation of change was calculated as:

$$\:\sqrt{{SD}_{pre}^{2}+\:{SD}_{post}^{2}-(2\:\times\:r\:\times\:\:{SD}_{pre}\:\times\:\:{SD}_{post}}$$

where r is the correlation between pre and post scores [40]. When the correlation of pre-post scores or the standard deviation of change was not presented in the text or could not be calculated, a conservative but also probable correlation effect size of *r* = 0.5 was used [41]. Where standard errors or confidence intervals were presented in the text rather than standard deviations, standard deviations were calculated [40]. The I-squared statistic was used to quantify between-study heterogeneity. The I-squared value is the proportion of variance in the meta-analysis due to between-study differences compared to random error within individual studies [42]. A higher value indicates that the results from the studies included in the meta-analysis are more heterogeneous [42].

#### Meta-Analysis

All meta-analyses were conducted using the meta package [43] in R v. 4.1.3 (R Core Team, Vienna, Austria) and R studio v. 1.3 (RStudio Team, Boston, MA). Pooled SMDs were estimated using a random effects meta-analysis and studies were weighted based on the inverse of their variance. The restricted maximum likelihood method was used to estimate between study heterogeneity. Given the inverse hypotheses, analyses for testosterone and SHBG were stratified by sex. Planned subgroup analyses were conducted to examine the difference in the effects of aerobic exercise on insulin sensitivity outcomes in participants with obesity or T2D separately.

## Results

**Fig. 1:**
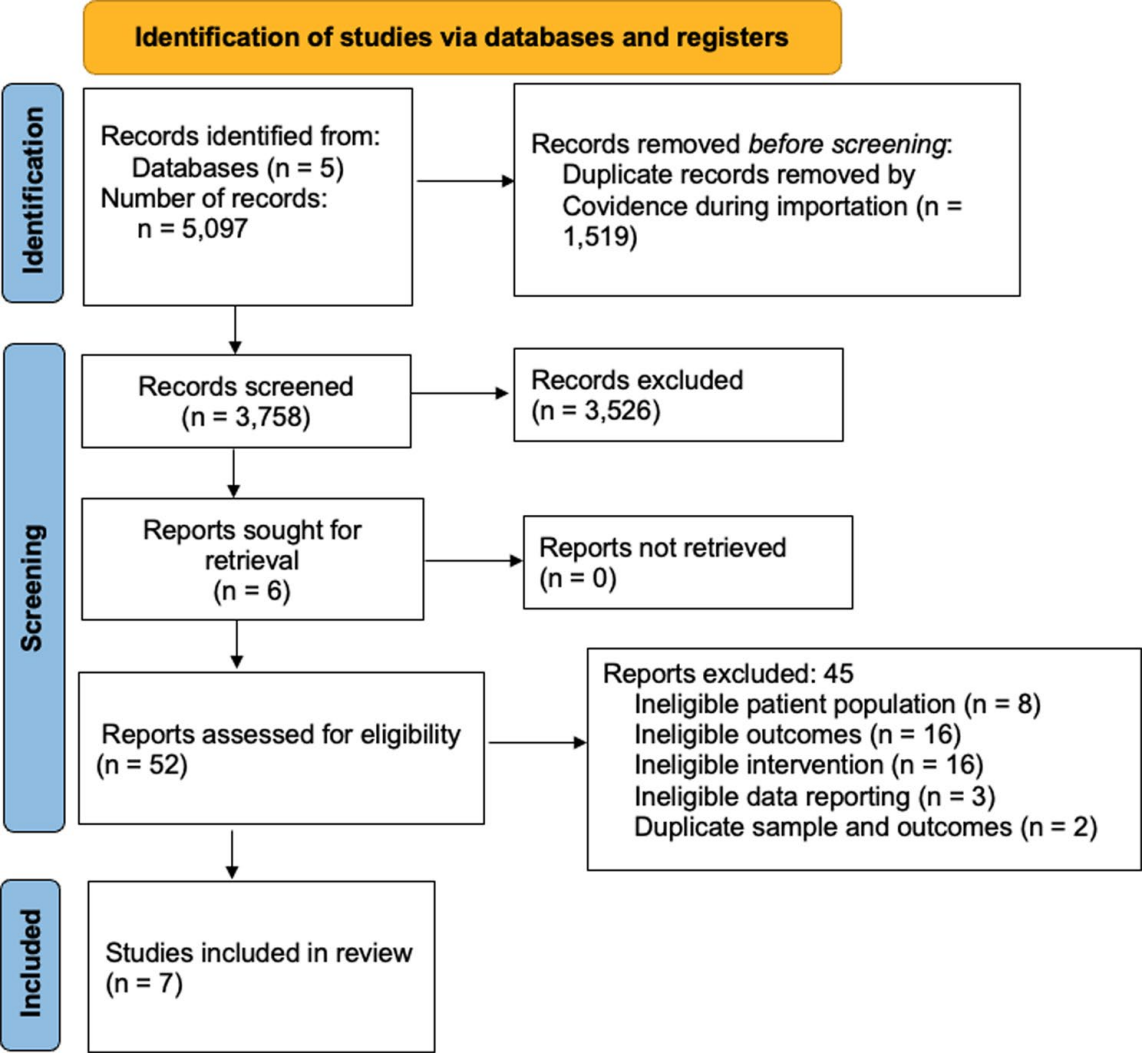
Preferred Reporting Items for Systematic reviews and Meta-Analyses (PRISMA) study selection flow diagram

The search strategy identified 5,097 papers for review (Fig. 1). Of these, 1,519 duplicates were removed. 3,578 underwent title and abstract screening with 3,526 excluded due to not meeting inclusion criteria. The remaining 52 articles underwent full-text screening for eligibility and 45 were excluded for reasons such as ineligible population, ineligible outcomes, ineligible intervention, ineligible study design, ineligible data reporting with no response from authors or duplicate sample and outcomes. The remaining seven studies were included in this systematic review and meta-analysis. Participant and study characteristics can be found in Table 2 and the exercise intervention and study outcomes can be found in Table 3.

**Table 2:** Participant characteristics

Study	Study design	Sample size	Participant sex	Population	Age (years)	BMI (kg/m^2^)
					Mean ± SD	Mean ± SD
Alkhatib et al., 2020 [44]	RCT	12	Female	Obese	21.7 ± 3	27.4 ± 3.9
Gonzalo-Encabo et al., 2020 [45]	RCT	10	Female	Obese	56.6 ± 0.8	32.3 ± 1.4
Kumagai et al., 2018 [37]	CT	28	Male	Obese	50 ± 6.3	27.4 ± 2.1
Onuora et al., 2022 [46]	CT	18	Male	Obese	29–47∋	30–39.9∋
Walker et al., 1999 [47]	CT	11	Female	T2D	58 ± 6	31.1 ± 5.6
Boudou et al., 2000 [48] (moderate and high intensity)	RCT	8	Male	T2D	42.9 ± 3.2	31.2 ± 1.1
Boudou et al., 2000 [48] (low intensity)		8			47 ± 7.2	28.3 ± 3.9
Hutchison et al., 2011 [49]	CT	8	Female	Obese	35 ± 8.3	30.8 ± 5.2

∋ Only range provided by author; RCT: randomised controlled trial; CT: controlled trial; T2D: type 2 diabetes; BMI: body mass index; SD: standard deviation

**Table 3:** Summary of studies identified for meta-analysis detailing participants, intervention characteristics and main outcomes measures

Study	Population	Exercise modality	Low, moderate, high intensity	Exercise intervention characteristics						Hormone outcomes				Insulin sensitivity outcomes	Changes in BMI, BF%, BW
				Duration (Weeks)	Session duration (Minutes)	Frequency Per week	Description	HR or VO_2_%	Supervised? Y/N/P	Testosterone			SHBG		
										Baseline (mmol/L)	Δ (mmol/L)	Change ↑/↓/↔			
Alkhatib et al., 2020 [44]	Obesity	HIIT	High	8	20	3	10 × 1 min cycling sprints, 1 min rest	85–95% HR_max_	Y	1.464	-0.20	↓	Not reported	FBG: ↔ HOMA-IR: ↔ Insulin: ↔	BMI: ↔ BF%: ↓
Gonzalo-Encabo et al., 2020 [45]	Obesity	SS	Moderate	12	60	3	Endurance training circuits, 10 min of treadmill, cycle-ergometer and elliptical machine	55–75% HRR	Y	0.99	-0.34	↓	↔	Only baseline data available	BMI: ↓ BF%: ↓
Kumagai et al., 2018 [37]	Obesity	SS	Low to Moderate	12	40–60	1–3	15–20 min warm up, approximately 40–60 min walking and/or light jogging, 15–25 min cooldown. Encouragement of M-VPA 150 min/week at home	Self-paced	P	15.4	+ 2.70	↑	↔	HbA1c: ↔ FBG: ↔ HOMA-IR: ↔ Insulin: ↓	BMI: ↓ BF%: ↓
Onuora et al., 2022 [46]	Obesity	SS	Moderate	12	35–50	3	10–15 min warmup, 35–50 min on a total cross bar machine	64–76% HR_max_	Not reported	11.44	+ 2.08	↑		Not reported	BW: ↓
Walker et al., 1999 [47]	T2D	SS	Low	12	60	5	Self-reported 60 min walk	Self-paced	N	1.33	-0.06	↔	↔	HbA1c: ↓ FBG: ↓ Insulin: ↔	BMI: ↓ BF%: ↓
Boudou et al., 2000 [48]	T2D	SS & HIIT	Moderate, High	8	25 min SS 45 min HIIT	3	SS: Continuous cycling exercise bout; HIIT: Cycling, 5 × 2 min, 3 min active recovery	SS: 75% VO_2peak_ HIIT: 85% & 50%VO_2peak_	Y	15.5	+ 1.35	↔	↔	HbA1c: ↓ FBG: ↔ Insulin: ↔	BMI: ↔
Boudou et al., 2000 [48]		SS	Low	8	20 min	1	Cycling, 30 W, 60 rpm	Not specified	Y	13.75	+ 3.45	↔	↔	HbA1c: ↔ FBG: ↔ Insulin: ↔	BMI: ↔
Hutchison et al., 2011 [49]	Obesity	SS & HIIT	Moderate, High	12	60	3	SS: 1 session of treadmill walking or jogging HIIT: 2 sessions, treadmill walking/jogging, 6 × 5 min bouts, 2 min recovery, progressing to 8 sets by week 4 and reduced recovery time to 1 min by week 8	SS: 75–85% HR_max_ HIIT: 95–100% HR_max_	Y	1.4	+ 0.40	↔	↔	FBG: ↔ HOMA-IR: ↓ Insulin:↔	BMI: ↔ BF%: ↓

SS: steady state; HIIT: high intensity interval training; HR: heart rate; VO_2_: oxygen: VO_2peak_: peak oxygen consumption; SHBG: sex hormone-binding globulin; FBG: fasting blood glucose; HOMA-IR: homeostatic model assessment for insulin resistance; BMI: body mass index; BF%: body fat percentage; BW: body weight; M-VPA: moderate to vigorous physical activity; W: watts; RPM: revolutions per minute; Y/N/P: yes/no/partially; Δ: delta change; HR_max_: maximum heart rate; HRR: heart rate reserve

### Summary of Articles

#### Study Design and Risk of Bias

Of the seven trials included in this systematic review and meta-analysis, three were RCTs [44, 45, 48], and four were CTs [37, 46, 47, 49]. One of the RCT studies prescribed their control group a different intensity of aerobic exercise training and was therefore included in the exercise analysis [48].

RoB for the RCTs included in this study were some concerns [48] and high risk of bias [44, 45] (Table 4). High risk of bias results were consequential to the use of immunoassays to quantify testosterone concentrations in women [44, 45]. ROBINS-1 for the CTs in this study were low risk [37, 47] and moderate risk [46, 49] (Table 5).

**Table 4:**
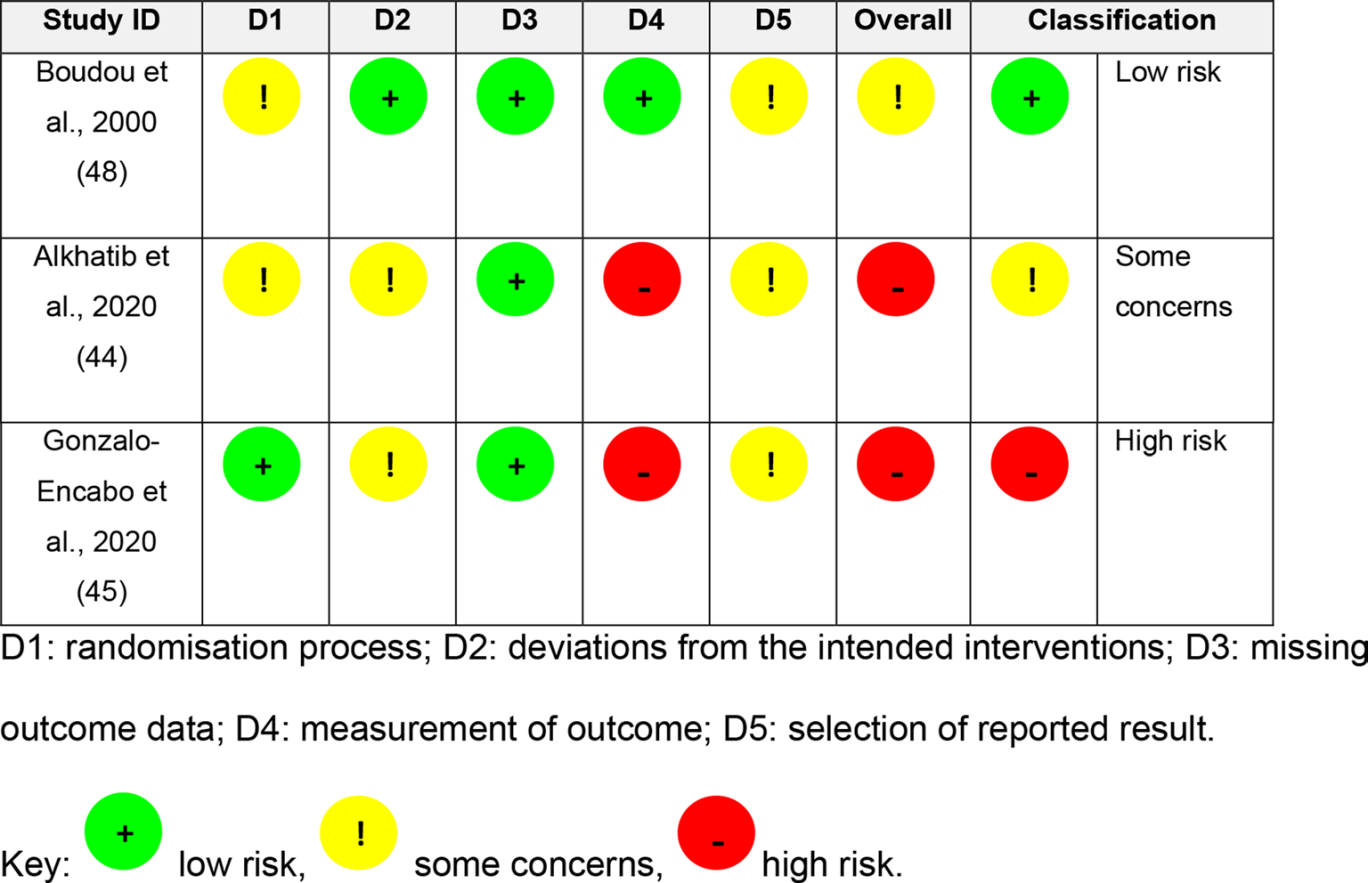
Risk of bias for randomised controlled trails

**Table 5:**
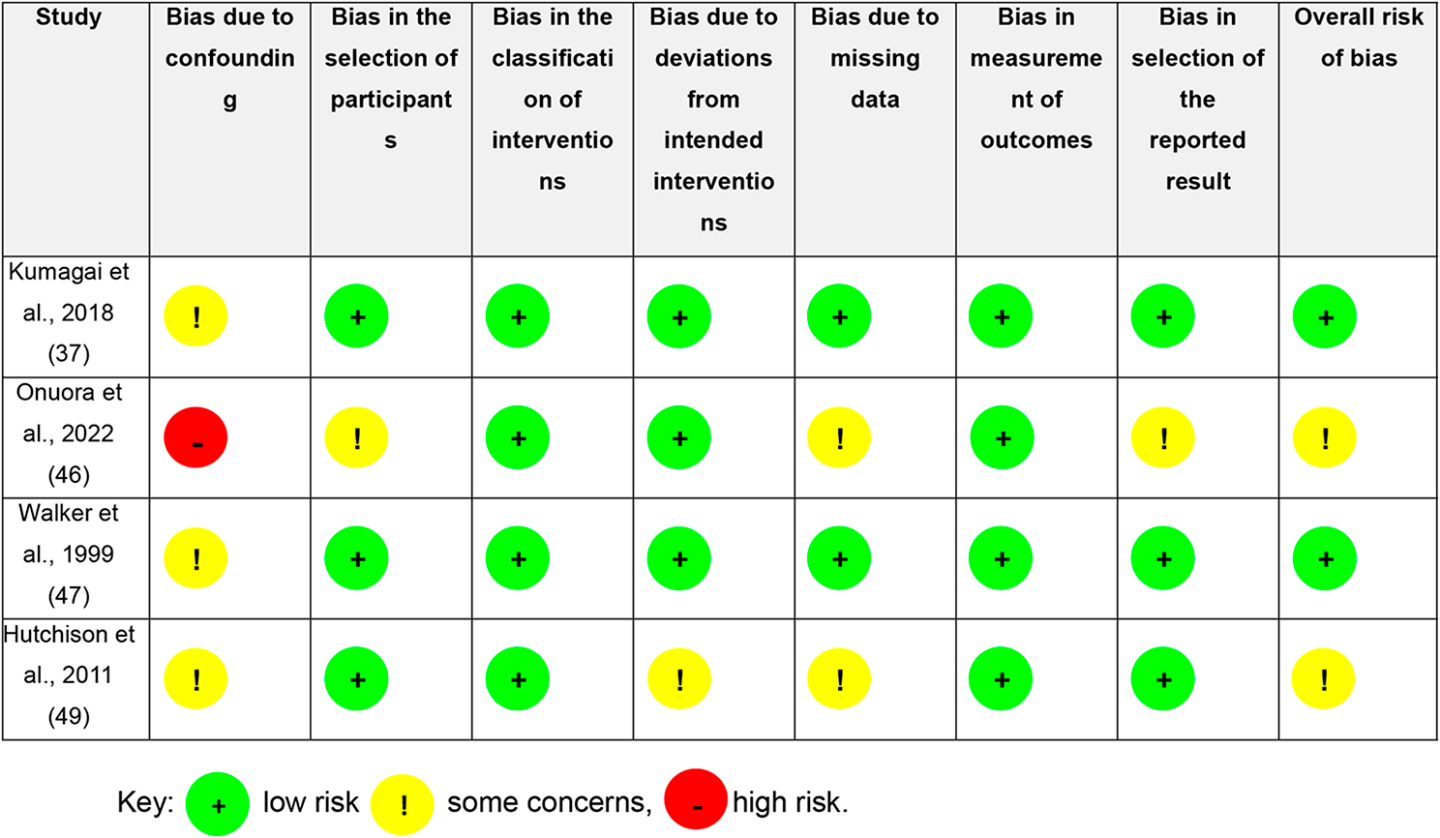
Risk of bias in non-randomised studies (ROBINS-I)

#### Participant Characteristics

The total participants included in this review was 103, of whom, 76 were obese and 27 had T2D. The mean age of participants ranged from 21 to 58 years and mean BMI ranged from 27.4 to 39.9 kg/m^2^. Study sample sizes ranged from eight to 28. Three studies were in men [37, 46, 48], and four were in women [44, 45, 47, 49]. Five studies investigated individuals who were obese [37, 44–46, 49]; two studies investigated individuals with T2D [47, 48].

#### Participant Randomisation or Group Allocation

Two RCT studies did not specify their randomisation method for participant allocation into either a control or aerobic exercise intervention group [44, 48]. One RCT study utilised a concealed method [45].

One CT study randomly selected a subgroup of participants with obesity to undertake an aerobic exercise intervention [46]. No information on randomisation method was described [46]. All other CT studies were allocated based on BMI or clinical condition (i.e., T2D, polycystic ovarian syndrome) [37, 47, 49].

#### Aerobic Exercise Intervention

Five studies implemented a steady state exercise intervention [37, 46–48]. Two studies used low intensity steady state with no specific working heart rate (HR) intensity [47, 48], one used low to moderate (self-paced) [37] and one used moderate intensity (64–76% [HR_max_]) [46]. One study utilised high-intensity interval training (HIIT) (85–95% HR_max_) [44], while another combined HIIT (85% peak oxygen consumption (VO_2peak_)) and moderate intensity steady state training (75% VO_2peak_) [48]. In terms of supervision, four studies had supervised exercise sessions [44, 45, 48, 49], one study was partially supervised (at least one supervised session per week) [37], another did not supervise the exercise sessions [47], and one did not report on supervision [46]. Finally, intervention duration and session duration ranged from eight to 12 weeks, and 20 to 60 min respectively. Frequency per week was one to five sessions.

### Systematic Review

Free testosterone, oestradiol, DHEA, FSH/LH and QUICKI were excluded from analysis as they were reported in ≤ 1 study [37, 44, 46, 48].

#### Sex Hormone and Insulin Sensitivity Outcome Analysis Method

Methods to analyse sex hormones and insulin sensitivity outcomes can be found in Table 6.

**Table 6:** Sex hormone and insulin sensitivity outcome analysis methods

Author name	Testosterone	SHBG	FBG	Fasting insulin	HbA1c
Alkhatib et al., 2020 [44]	ELISA (IBL America, Minneapolis, MN)	N/A	Portable analyser (Accu-chek)	ELISA (Mercodia, Uppsala, Sweden)	N/A
Gonzalo-Encabo et al., 2020 [45]	CLIA (Centauro XP, Siemens Healthineers)	Solid phase “sandwich” IRMA (Cisbio Bioassays)	Method not reported		
Kumagai et al., 2018 [37]	CLIA (LSI Medience, Ibaraki, Japan)	ELISA (DSHBG0; R & D)	*Standard enzymatic techniques		
Onuora et al., 2022 [46]	ELISA (Mindray, MR-96 A)	N/A			
Walker et al., 1999 [47]	RIA (Orion Diagnostica, Espoo, Finland)		Automated enzymatic methods*	RIA (Orion Diagnostica, Espoo, Finland)	Ion-exchange chromatography*
Boudou et al., 2000 [48]	RIA (bioMérieux, Lyon, France)		Glucose oxidase method	RIA (INSIK-5 kit, Sorin Biomedia, Antony, France)	Method not reported
Hutchison et al., 2011 [49]	Automated competitive binding immunoenzymatic assay (Beckman Coulter Diagnostics Australia, Gladesville, Australia)	Automated enzyme immunoassay (Diagnostic Products Corp., Los Angeles, CA)	Commercial enzymatic kits (Beckman Coulter Diagnostics Australia, Gladesville, Australia)	RIA (Linco Research, St. Charles, MO)	N/A

*As described by author. N/A: not applicable; ELISA: enzyme-linked immunosorbent assay; CLIA: chemiluminescent immunoassay; IRMA: immunoradiometric assay; RIA: radioimmunoassay

#### Testosterone

Two studies included in this review reported a significant increase in testosterone concentrations following low to moderate, and moderate intensity aerobic exercise training in men who were obese [37, 46]. The third study in this review which included men with T2D reported no significant changes in testosterone concentrations following low, moderate and high intensity training in two aerobic exercise intervention groups [48].

One study reported a decrease in testosterone concentrations following moderate intensity training and HIIT in women who were obese [44, 45], while another found no changes in testosterone concentrations following moderate intensity training and HIIT [49]. In women with T2D, no changes in testosterone were reported following a low intensity aerobic exercise intervention [47].

#### SHBG

Of the seven studies included in this review, four studies reported SHBG [37, 45, 47, 48]. All studies, regardless of sex, metabolic status (obese or T2D) or exercise intervention, independently reported no significant changes in SHBG [37, 45, 47, 48].

#### Insulin Sensitivity Outcomes

Six studies included in this review reported FBG [37, 44, 47–49], but, one study only reported baseline values [45]. Four studies reported no change in FBG following aerobic exercise training [37, 44, 48, 49]. One study reported a significant decrease in FBG following low intensity aerobic exercise training in women with T2D [47].

Three studies included in this review reported on HOMA-IR. One study reported a significant decrease in HOMA-IR following moderate intensity training and HIIT in women who were obese [49], while two studies, one study reporting on either sex, found no changes in HOMA-IR following low to moderate [37] and HIIT [44].

A total of six studies included in this review reported fasting insulin [37, 44, 47, 48, 50], but, one study only reported baseline values [45]. Four studies, including one study with two intervention groups of participants with T2D, reported no changes in insulin [47–49]. One study in men who were obese reported a significant decrease in insulin following low to moderate aerobic exercise training [37].

Of the seven studies included in this review, four reported HbA1c [37, 47, 48]. One study only reported baseline values [45]. In men with T2D, a decrease in HbA1c levels was reported following moderate intensity training and HIIT, but no change was found following low intensity [48]. A single study in women with T2D reported a significant decrease in HbA1c following low intensity exercise [47]. Finally, one study in men who were obese reported no change in HbA1c following low to moderate intensity aerobic exercise [37].

### Meta-Analysis

The results from this meta-analysis investigating the effects of aerobic exercise on testosterone, and other sex hormone and insulin sensitivity markers, are detailed below.

#### Testosterone

**Fig. 2:**
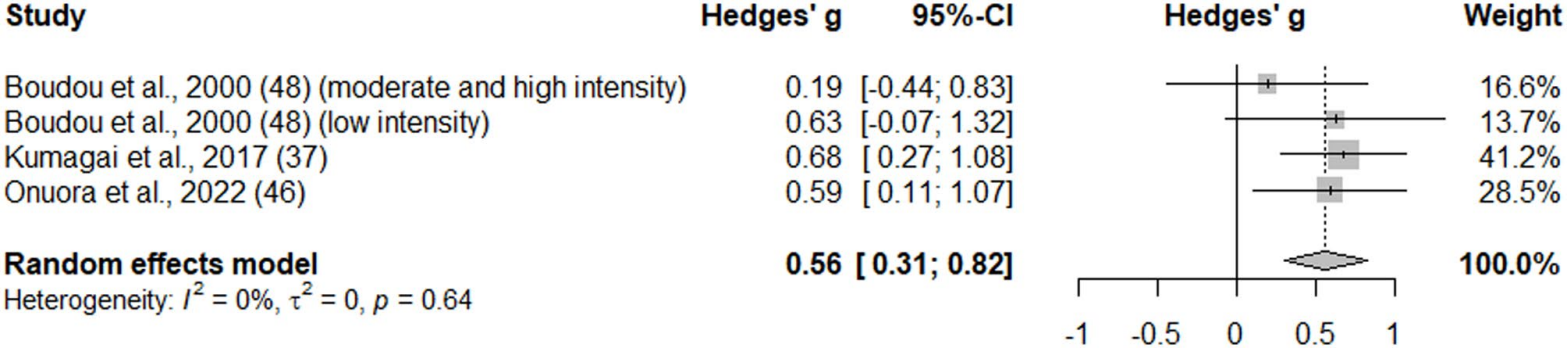
Forest plot of the standardized mean differences (SMD) of testosterone concentrations following aerobic exercise intervention in men who are obese or have T2D. A negative value indicates a reduction of the outcome after aerobic exercise training, whereas a positive value indicates an increase of the outcome after aerobic exercise training. Abbreviations: CI, confidence interval.

The current review included three studies and four intervention groups in men with a total sample size of 62 (46 with obesity, 16 with T2D) [37, 46, 48] (Fig. 2). Serum total testosterone concentrations were significantly higher following aerobic exercise intervention (g = 0.565, 95% CI = 0.307 to 0.822, *p* < 0.001, I^2^ = 0.0%). 

**Fig. 3:**
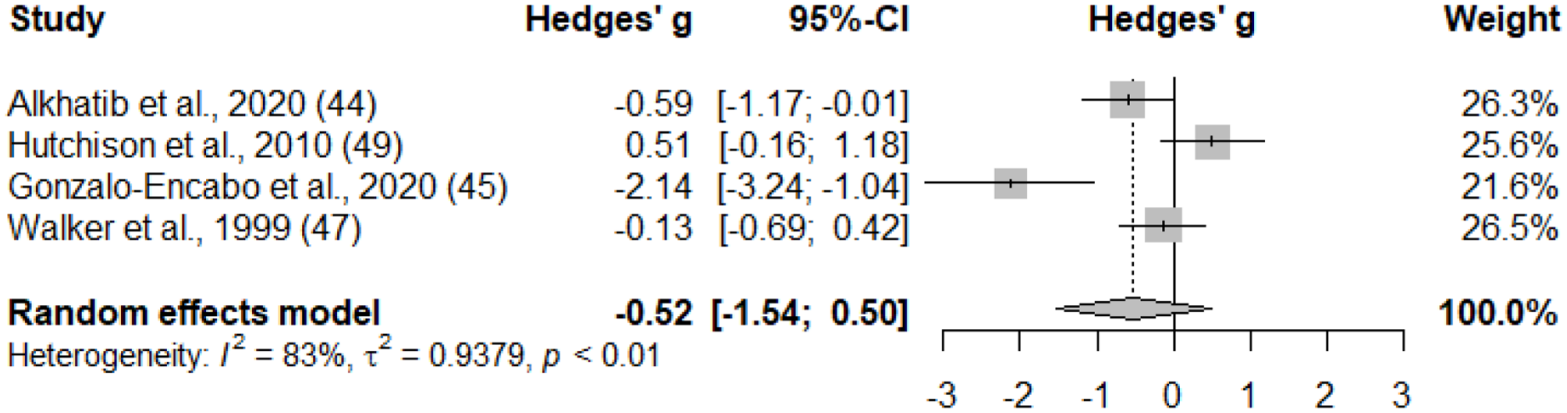
Forest plot of the standardized mean differences (SMD) in testosterone concentrations following aerobic exercise intervention in women who are obese or have T2D. A negative value means a reduction of the outcome after aerobic exercise training, whereas a positive value means an increase of the outcome after aerobic exercise training. Abbreviations: CI, confidence interval

Four studies in women, which included a total of 41 participants (30 with obesity, 11 with T2D), were analysed for changes in serum total testosterone concentrations post aerobic exercise intervention [44, 45, 47, 49] (Fig. 3). No significant changes in testosterone concentrations were found (g = -0.523, 95% CI = -1.541, 0.496, *p* = 0.314, I^2^ = 83.0%).

#### SHBG

Meta-analysis of two studies in men, which included a total of 44 participants (28 with obesity, 16 with T2D), total intervention groups *n* = 3, and two studies in women who were obese with a total of 19 participants, were analysed for the effects of aerobic exercise training on SHBG [37, 45, 47, 48]. The results showed no significant changes in SHBG in men (g = -0.027, 95% CI= -0.307 to 0.252, *p* = 0.848, I^2^ = 0.0%) or women (g = 0.091, 95% CI -0.315 to 0.513, *p* = 0.639, I^2^ = 0.0%).

#### Insulin Sensitivity Outcomes

Five studies included in the meta-analysis accessed the effects of aerobic exercise on FBG [37, 44, 47–49]. These included a total of 75 participants (44 men / 31 women, 48 with obesity, 27 with T2D). No significant changes in FBG were found (g = -0.093, 95% CI = -0.311 to 0.124, *p* = 0.400, I^2^ = 17.2%). Subgroup analysis of individuals who are obese (g = -0.064, 95% CI = -0.336 to 0.208, *p* = 0.645, I^2^ = 0.0%) and individuals with T2D (g = -0.139, 95% CI = − 0.0647 to 0.369, *p* = 0.592, I^2^ = 49.7%) also showed no significant changes in FBG following aerobic exercise training.

Meta-analysis of HOMA-IR was conducted on three studies, with a total of 48 participants (28 men / 20 women, all with obesity) [37, 44, 49]. The results showed no significant changes in HOMA-IR following an aerobic exercise intervention (g = -0.174, 95% CI = -0.450 to 0.102, *p* = 0.216, I^2^ = 26.5%).

Five studies, with a total of 75 participants (44 men / 31 women, 48 with obesity, 27 with T2D), were included in the meta-analysis on fasting insulin and aerobic exercise [37, 44, 47, 48, 50]. The results showed no significant changes to insulin following aerobic exercise training (g = -0.098, 95% CI = -0.346 to 0.149, *p* = 0.437, I^2^ = 12.3%). Subgroup analysis of individuals who are obese (g = -0.231, 95% CI = -0.492 to 0.030, *p* = 0.082, I^2^ = 38.5%) and individuals with T2D (g = 0.069, 95% CI = -0.277 to 0.416, *p* = 0.695, I^2^ = 0.0%) also showed no significant changes in insulin following aerobic exercise training.

Three studies, which included a total of 55 participants (44 men / 11 women, 28 with obesity, 27 with T2D), were included in the meta-analysis on HbA1c and aerobic exercise [37, 47, 48]. No significant changes were observed in HbA1c following aerobic exercise training (g = -0.439, 95% CI = -1.144 to 0.267, *p* = 0.223, I^2^ = 74.9%). Subgroup analysis of individuals with T2D also showed no significant changes in HbA1c following aerobic exercise training (g = -0.533, 95% CI = -1.529 to 0.462, *p* = 0.294, I^2^ = 77.9%).

## Discussion

To our knowledge, this is the first systematic review and meta-analysis to investigate the effects of aerobic exercise training on total testosterone concentrations in men and women who are obese or have T2D. Overall, we report that aerobic exercise training can be used as a tool to increase total testosterone concentrations in men, but, due to the limitations within the current data, it remains unclear whether aerobic exercise can significantly influence testosterone concentrations in women. In addition, aerobic exercise training interventions between 8 and 12 weeks in duration do not appear to significantly influence SHBG or markers of insulin sensitivity in men and women who are obese or have T2D.

Results from this meta-analysis suggest that aerobic exercise training can induce moderate increases in testosterone concentrations in men, and, within the limitations of the current data, does not appear to significantly influence testosterone concentrations in women. Previous literature is in support of our findings, reporting significant increases in testosterone concentrations following an aerobic exercise intervention in men, but not in women, who are obese [51–53]. However, it should be noted that for women, a large majority of studies utilise immunoassays to quantify testosterone, including those in this review [45, 49]. Immunoassays have been deemed suboptimal for analysis of testosterone in women as they can have poor sensitivity and precision when detecting low concentrations of testosterone, such as that found in a female population [54]. Thus, the current findings in women may not be reliable. Nonetheless, the current results provide an indication of efficacy of aerobic exercise training for improving total testosterone concentrations in women who are obese.

We identified only one study in individuals with T2D for each sex and neither reported significant changes in testosterone concentrations post exercise [47, 48]. However, due to the smaller sample size, as well as one study being a self-paced and self-reported exercise intervention with the use of immunoassay for testosterone quantification in women [47], it remains unclear how or if aerobic exercise can influence testosterone in a population with T2D. Given that many individuals who are obese progress to T2D [55], it is unlikely that exercise ceases having any effect on testosterone concentrations once IR becomes prevalent, but this has yet to be demonstrated and further studies are required to test this hypothesis.

High exercise volumes and low energy availability have been associated with hypogonadism in both men and women [56, 57]. This suggests that, though contrasting, both states i.e., sedentary with high energy availability vs. athletic with low energy availability, result in suppression of the hypothalamic pituitary gonadal (HPG) axis. It is therefore likely an optimal exercise dose exists for desirable hormone outcomes.

Indeed, studies included in this review reported no changes in testosterone concentrations in men following 8 weeks of low or moderate intensity exercise, or HIIT [48]. However, significant increases were reported following 12 weeks of exercise [37, 46]. Intervention variables, such as intensity, frequency, and session duration, were not dissimilar between studies [37, 46, 48], suggesting a potential tipping point between 8 and 12 weeks for positive improvements in testosterone concentrations in men. However, no trend between exercise intervention and testosterone changes were identified in women, with no change or decreases in testosterone concentrations reported for similar intervention characteristics and similar baseline testosterone concentrations [44, 45, 47, 49]. Thus, while we can suggest that aerobic exercise training characteristics, such as intensity and duration, may play a role in testosterone responses to exercise, we have insufficient evidence to fully elucidate optimal exercise dosage for desirable hormone changes in individuals who are obese or have T2D and this should be explored further in future studies.

Previous literature has demonstrated that obesity is a stronger risk factor for low testosterone concentrations in men than T2D [58], and that weight loss can result in increased testosterone concentrations in men who are obese [19]. Though the exact mechanisms are unclear, it has been proposed that the metabolic consequences to excess body fat, such as increased pro-inflammatory adipocytokines, impaired insulin and dysregulated leptin signalling, contribute to hypothalamic suppression, consequently reducing HPG axis activity and sex hormone production [58, 59]. Thus, decreases in excess body fat mass may be beneficial for the HPG axis and should be considered alongside an exercise intervention for desirable hormonal changes. For instance, studies in men included in this review that reported a significant decrease in anthropometric measurements, i.e., BMI, body fat percentage, body weight, also reported a significant increase in testosterone concentrations [37, 46]. This relationship was not identified in women [44, 45, 47, 49], but, as previously stipulated, immunoassay use may have influenced these results. Indeed, previous researchers who utilised sensitive and specific radioimmunoassays to quantify testosterone have reported that women who lose > 2% body fat following three months of aerobic exercise have a subsequent 10.1% decline in total testosterone concentrations [52]. Such a decline was not observed in exercisers who maintained or increased their body fat percentage during the intervention, nor was it observed in the control group [52]. Thus, the lack of change observed in women who lost body fat may have been due to the use of immunoassays to analyse testosterone.

In addition, high insulin levels as a result of obesity and/or T2D are associated with decreased SHBG synthesis [60], resulting in higher unbound and active sex hormones [61]. In women, this elevation in free sex hormone concentrations has been associated with higher androgen production [59, 62]. In men, however, changes to testosterone and SHBG appear to occur in parallel, resulting in constant free testosterone concentrations [19, 63]. Thus, based on the current knowledge, aerobic exercise in conjunction with fat loss may be superior to exercise alone for improving testosterone concentrations in both men and women, however, further research is required.

This meta-analysis found no significant changes in SHBG levels following aerobic exercise training in men and women who are obese or have T2D [37, 45, 47, 48]. This lack of change in SHBG was consistent regardless of changes in BMI or body fat percentage [37, 45, 47, 48] or insulin levels reported by the studies [37, 49]. Campbell et al., 2012 [53] reported contrasting findings. While they also reported no significant change in SHBG levels following 52 weeks of moderate/high intensity aerobic exercise training in women who were obese, they did report that weight loss of > 5% incurred a significant increase in SHBG, regardless of assigned group (exercise, diet, or diet + exercise) [53]. Similarly, Niskanen et al., 2004 [64] reported that in men who are obese, fat loss following a very low calorie diet resulted in a 43% increase in SHBG [64], suggesting that, similar to testosterone, fat loss plays a significant role in SHBG changes. As individuals who are obese or have T2D have lower levels of SHBG [65, 66] and emerging evidence suggests that SHBG may be an indicative marker of cardiometabolic disease risk, including risk of IR, obesity, T2D and other metabolic disorders [10, 67, 68], the influence of exercise on SHBG and its association with anthropometric measures should be further investigated. Additionally, as studies included in this review reported no changes in SHBG, but increases in testosterone following aerobic exercise training in men, this may suggest that aerobic exercise can increase free testosterone concentrations in men. Thus, future studies should investigate the effects of aerobic training on testosterone, free testosterone and SHBG in men who are obese or have T2D.

Our meta-analysis found no significant changes in insulin sensitivity outcomes following aerobic exercise in men and women who are obese or have T2D. While some of the reviewed studies independently reported significant decreases in FBG [47], HOMA-IR [49], insulin [37, 49] and HbA1c [47, 48] following 8 or 12 weeks of self-paced aerobic exercise training or HIIT [37, 47–49], other studies reported no changes following similar exercise interventions [37, 44, 47–49]. Therefore these findings may have been influenced by the small sample sizes within the independent studies as well as the limited number of studies that include both testosterone and insulin sensitivity markers following aerobic exercise in populations that are obese or have T2D.

Though some studies have also reported no changes in insulin sensitivity markers following aerobic exercise [69, 70], the predominant literature supports that aerobic exercise, and/or general physical activity, improves insulin sensitivity outcomes, including FBG, HOMA-IR, insulin and HbA1c [71–76]. Thus, the lack of change observed here may be due to the small sample size included in this meta-analysis.

The meta-analysis has some potential limitations including a small sample size for each sex and metabolic condition (obesity and T2D), as well as the inclusion of studies utilising immunoassays to quantify testosterone in women which can have poor precision. Analysis based on metabolic condition (obesity vs. T2D) was not conducted due to the small number of studies available, particularly for individuals with T2D. This study highlights the need for further research on testosterone and exercise in people at risk of, or with, T2D as it is an important research area. Future studies and future meta-analyses should separate these groups once more data are available. In addition, due to the limited literature, we were unable to compare changes in testosterone concentrations following an aerobic exercise intervention to a standard care control group that is obese or has T2D. Future studies with larger sample sizes and appropriate analysis methods for testosterone concentrations in women are required. Further, T2D is often listed a comorbidity in research and this can make paper identification and data extraction difficult. Future studies should conduct sub-analyses for participants with T2D, particularly given its increasing global presence. Finally, testosterone and other sex hormones are often not primary research outcomes in papers and are consequently only listed in tables or figures. This makes identifying papers for review difficult, particularly in the context of the PRISMA guidelines. Articles that analyse sex hormones should mention results in the text so that they can be identified by future studies using keyword searches.

## Conclusion

Aerobic exercise training may be used to increase total testosterone concentrations in men who are obese or have T2D, but, within the limitations of the current data, does not appear to significantly influence testosterone concentrations in women. Given the low number of studies, further studies investigating the effect of exercise on circulating sex hormones in men and women who are obese or have T2D are needed. In addition, given the close association of low testosterone and obesity/diabetes, further studies should evaluate whether exercise-induced alterations in circulatory testosterone mediate some of the salutary effects of exercise.

## Data Availability

The datasets used and/or analysed during the current study are available from the corresponding author on reasonable request.

## Abbreviations

T2D: Type 2 diabetes
IR: Insulin resistance
PICOS: Population, Intervention, Comparison, Outcomes and Studies
BMI: Body mass index
DHEA: Dehydroepiandrosterone
FSH: Follicle stimulating hormone
LH: Luteinising hormone
FBG: Fasting blood glucose
QUICKI: Quantitative insulin-sensitivity check index
HOMA-IR: Homeostatic model assessment for insulin resistance
HbA1c: Hemoglobin A1c
RoB2: Risk of Bias 2 assessment tool
RCT: Randomised controlled trial
ROBINS-I: Risk Of Bias In Non-randomised Studies – of Interventions
CT: Controlled trials
SMD: Standardised mean difference
VO2peak: Peak oxygen consumption
HIIT: High-intensity interval training
HR_max_: Maximum heart rate
HPG: Hypothalamic pituitary gonadal
SD: Standard deviation
SS: Steady state
HR: Heart rate
BF%: Body fat percentage
BW: Body weight
M-VPA: Moderate to vigorous physical activity
PRISMA: Preferred Reporting Items for Systematic reviews and Meta-Analyses
ELISA: Enzyme-linked immunosorbent assay
CLIA: Chemiluminescent immunoassay
IRMA: Immunoradiometric assay
RIA: Radioimmunoassay
